# A Simple Hot-Pressing Strategy for Thick Lithium Iron Phosphate Electrodes with Outstanding Electrochemical Properties

**DOI:** 10.1021/acsomega.5c06687

**Published:** 2025-12-29

**Authors:** Antonio J. Fernández-Ropero, Daniel del Rio-Santos, Belén Levenfeld, Alejandro Varez

**Affiliations:** Department of Materials Science and Engineering and Chemical Engineering, IAAB, 16726 Universidad Carlos III de Madrid, Madrid, Leganes 28911, Spain

## Abstract

The growing demand for electric vehicles and renewable energy storage has intensified the need for Li-ion batteries with higher energy density. One effective strategy is the use of high mass loading electrodes, which increase the ratio between active and inactive materials. However, conventional tape casting techniques face challenges in producing thick electrodes as mechanical consistency deteriorates beyond a certain thickness. Alternative methods have been explored but often require changes in additives or compromise electrochemical performance. In this work, we present a simple and scalable modification of the traditional electrode fabrication process. By drying the NMP solvent before pressing and applying low-temperature hot pressing (190 °C), we obtain thick (150–650 μm), homogeneous, and mechanically robust electrodes that retain the use of standard additives such as carbon black and polyvinylidene fluoride (PVDF). A full cell composed of LFP/LP30/LTO delivered outstanding results: high areal capacities (17 and 13.5 mAh cm^–2^ at C/25 and C/4), and exceptional cycling stability with no capacity loss over 300 cycles at C/12, despite a high loading of 120 mg cm^–2^ (650 μm). This approach requires minimal changes to current industrial processes, offering a promising route for next-generation Li-ion batteries with improved energy density and performance.

## Graphical abstract

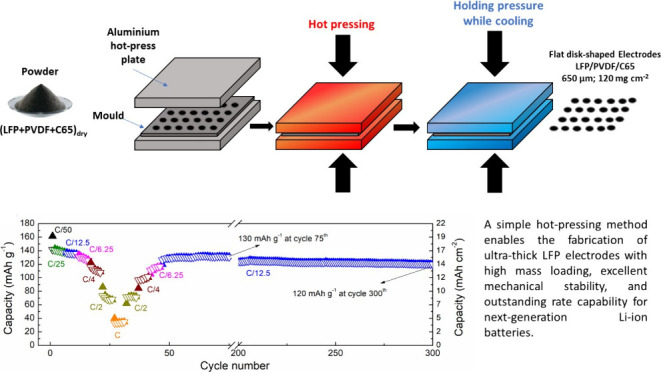

## Introduction

There is a growing interest in the development of thick electrodes with a reduced amount of inactive material (collectors and separators) that do not contribute to energy storage, thus lowering production costs and increasing the autonomy of the battery.^1,2^ However, thicknesses above 150 μm suffer from delamination and a loss of consistency during processing by the conventional tape casting method. This limit is known as the critical cracking thickness (CCT), and it has been widely accepted that the capillary stresses during the drying process are the cause.^3^

In recent years, techniques avoiding the drying step have gained interest for the development of thick electrodes. Some methods can produce robust binder-free thick electrodes after a sintering step of pieces created by powder extrusion molding,^4^ additive manufacturing (3D printing, fused filament fabrication,^5^ or robocasting^6^), or cold pressing.^7^ Although the sintering step densifies the electrodes, reaching a higher mass loading per area,^8^ it removes any trace of binder and blocks the use of conductive additives. The lack of binder additive implies a higher vulnerability toward cracking and particle detachment due to volume changes produced upon Li^+^ insertion and deinsertion, eventually affecting long cycling performance.^9^ On the other hand, the absence of carbon diminishes the paths for electrons, reducing the electrical response at high rates.^10^ Carbon facilitates the conductivity of charged species in the cathode materials and helps disperse the negative charge accumulation, which may otherwise impede Li-ion diffusion within the electrode.^11,12^ Moreover, the carbon in conventional electrodes also influences the porosity of the electrode and works as a reservoir of electrolyte that facilitates the Li^+^ transport along the length of the electrode.^13^ Therefore, sintered electrodes have poor performance at high current densities due to lower ionic and electronic conductivity.

For those reasons, the development of techniques able to produce thick electrodes with additives is attracting interest. In this regard, Hu et al. developed electrodes containing LFP and LTO, super P, and PVDF supported by 3D porous conductive textiles. They effectively demonstrated electrodes with a mass loading of ∼168 mg/cm^2^ and a thickness of ∼650 μm, which are 8–12 times higher than those cast on the metal collector. The LFP-LTO full cell has an areal capacity of 21 mAh cm^–2^ at C/10, with a capacity retention of 88.5% after 30 cycles. Wang et al. developed freestanding electrodes with vertically aligned channels prepared by a phase inversion method.^14^ Electrode layers were made from an *N*-methyl-2-pyrrolidone (NMP) solution containing carbon additives and polyvinylpyrrolidone (PVP) and polyvinylidene fluoride-*co*-hexafluoropropylene (PVDF-HFP). Different thicknesses were obtained by overlapping layers. Electrodes of up to 1.3 mm thickness were shown, but with a mass loading of 100 mg cm^–2^, which implies a porosity near 70% that would drastically reduce the volumetric energy density of the cell. An areal capacity of 15 mAh cm^–2^ was reached at C/10, but the cycling stability was shown only at the high rate of 1C for 50 cycles with significant capacity fading.^14^ Similarly and more recently, freestanding LFP cathodes with vertically aligned microchannels (9.5 mg cm^–2^) prepared by freeze-drying showed 158.6 mAh g^–1^ with 96.3% retention after 250 cycles at 0.5C, while integration with in situ polymerized PDOL gel electrolyte delivered 132.5 mAh g^–1^ after 100 cycles with reduced polarization.^15^ A 3D-printing approach was also reported to design LFP electrodes containing CNT and PVDF-HFP, but even for a thickness of 1.5 mm, the areal capacity was limited to 7.5 mAh cm^–2^.^16^

One processing technique that can produce thick and consistent electrodes with no sintering at high temperatures is low-temperature hot pressing (LTHP), attractive for its easy application and potential scalability. LTHP has been extensively applied for polymeric solid electrolytes; however, the research on electrode fabrication is less frequent.^17^ Apart from allowing additives, a great advantage of LTHP is to be able to adjust the pressure to obtain different degrees of porosity, directly influencing the electrochemical performance.^17^ This method has been implemented using polymeric binders different from PVDF with more modest structural, thermal, or chemical properties (i.e., PEO or PTFE).^18,19^ These works used low-mass loading electrodes (>15 mg/cm^2^). A more recent publication by Kim et al. showed the performance of thick electrodes of 250 μm and 40 mg cm^–2^ using a phenoxy resin as binder.^20^ Although the cycling stability was superior to an electrode prepared by tape casting with the same dimensions, it was limited to 73.5% over 50 cycles at 0.1C. Similarly, Wu et al. prepared dense electrodes for an LCO/LTO solid-state cell by cold pressing a composite mixed in water and formed by active material and *LLZGO-PEO-C* (LLZGO is used to compensate for the low penetration of the organic electrolyte).^21^ Electrodes with thicknesses from 300 to 1500 μm were developed, showing fair capacity in lithium half-cells, but only those of 300 and 400 μm had stable cycling with 10 and 15 mAh cm^–2^, respectively.

In the present work, thick electrodes (650 μm) containing LiFePO_4_, carbon, and PVDF have been prepared by hot pressing at low temperature of a dry mixture of active material, carbon black, and PVDF that was previously mixed with common *N*-methyl pyrrolidone to favor the homogenization of the binder, the carbon black, and the active material (LFP and LTO). PVDF is preferred over other polymers due to its electrochemical stability, high adhesion to the current collector, and good electrolyte uptake.^22,23^ After drying and hot pressing the composite, ultrathick electrodes with a mass loading of 120 mg cm^–2^ and 650 μm were developed, displaying a superior electrochemical performance compared to other reported thick electrodes.

## Experimental Section

### Preparation and Characterization of LFP and LTO Electrodes

Commercial carbon-nanotube Li_4_Ti_5_O_12_ (CNT-LTO) and LiFePO_4_ (LFP) powders were supplied by Linyi Gelon LIB Co. LFP powder is supplied with a carbon coating film (1.70 wt % C measured with a LECO) and CNT-LTO with nanotubes (1.14 wt % C). For each material, a slurry containing the active material, PVDF, and C65 was mixed overnight in an *N*-methyl-2-pyrrolidone (NMP) solution. The active material-C65-PVDF ratio was 89/8/3 (wt.%), using 2.5 mL per gram of active material. Then, the NMP is dried by heating at 100 °C while stirring until complete evaporation. The obtained composite was pressed in a Fontijne heated platen press at 320 MPa and 190 °C for 30 min assisted by a circular mold with diameters from 10 to 14 mm and a thickness between 150 and 650 μm. A scheme of the process appears in Figure 1. The final thickness slightly increased after press decompression (up to 650 μm in the case of 500 μm). The microstructure of the sample was observed by using a field-emission scanning electron microscope (Teneo, FEI USA) with a secondary electron detector and an acceleration voltage of 10 kV. X-ray mapping images were collected by using the EDS EDAX TEAM analysis system (10 kV; 1.6 nA). The density of the prepared pieces was measured through the Archimedes method, employing acrylic lacquer as the sealing agent. Five samples were measured, and the average value is reported together with the standard deviation. The electrical conductivity of the samples was evaluated by impedance spectroscopy using an SI1260 impedance/gain-phase analyzer coupled to an SI1287 interface (Solartron). Measurements were performed at room temperature by applying a 10 mV amplitude signal in the 0.1 Hz–1 MHz frequency range. The samples were placed between two gold disks that served as ion-blocking electrodes.

**Figure 1:**
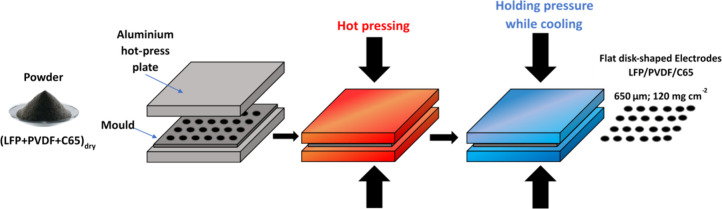
Schematic illustration of the fabrication process used in this work to prepare thick electrodes by a low-temperature flexible hot pressing. The figure highlights the main steps of the method.

### Electrochemical Characterization Performance

First, LFP electrodes were evaluated vs metallic Li in two-electrode stainless steel coin cells (type CR2032). Afterward, to avoid short circuits caused by dendrite formation, Li metal was replaced by CNT-LTO electrodes with a LFP:CNT-LTO of 1:2. The cells were assembled in a dry Ar-filled glovebox (<1 ppm of H_2_O and O_2_). The electrolyte was a solution of LP30 (1 M LiPF_6_ in EC/DMC = 50/50 (v/v), battery grade, Sigma-Aldrich) soaked in a glass fiber used as the separator. C-rates were calculated based on the full theoretical specific capacity of LFP (171 mAh·g^–1^ for 1 Li^+^ deintercalation/intercalation). LFP vs Li were cycled in the range 2.5 to 4 V. LFP-LTO cells were cycled in the voltage range of 1 to 2.6 V.

## Results and Discussion

The prepared LFP electrodes had a thickness of 650 μm and 120 mg cm^–2^, which corresponds to a density of 1.85 ± 0.09 g cm^–3^ (porosity of 45%) as determined with the Archimedes’ method density measurements. The presence of abundant pores is observable by SEM (Figure 2a). The electronic conductivity of the electrodes was evaluated as a function of thickness (150–650 μm). The results (Figure S2, Supporting Information) show that although the overall conductivity decreases for the thickest electrode due to increased porosity and longer transport pathways, the frequency-independent behavior of the admittance confirms that percolation of the conductive network is maintained throughout the electrode. The cells were first tested *vs.* metallic Li at very low C-rates with the purpose of determining if the electrochemical reaction takes place along the whole length of the electrode. The capacity at the first cycle at C/50 was 165 mAh g^–1^, near the theoretical capacity of 171 mAh g^–1^, which implies that the electrode is almost fully effective despite the high thickness. The overpotential was only 60 mV. For the next cycles, the rate was increased to C/25 (Figure 2b). The overpotential slightly increased to 78 mV and the specific capacity did not change. However, after a total of four charge/discharge cycles, the set charging potential could not be reached (Figure 2c). It can be explained by the formation of dendrites when Li^+^ ions are deposited on the Li foil that passes through the separator, producing an electrical short-circuit (Figure 2d,e). This exacerbated dendrite growth is produced by the high current density per unit area using thick electrodes with six times the loading of commercial typical electrodes.^24^ Additional cycling tests with a restricted discharge cutoff voltage of 3.0 V were also performed, and the results (Figure S3, Supporting Information) confirmed that dendritic growth and short-circuit still occur under these conditions, indicating that the phenomenon is mainly associated with Li deposition during charging rather than the discharge window.

**Figure 2:**
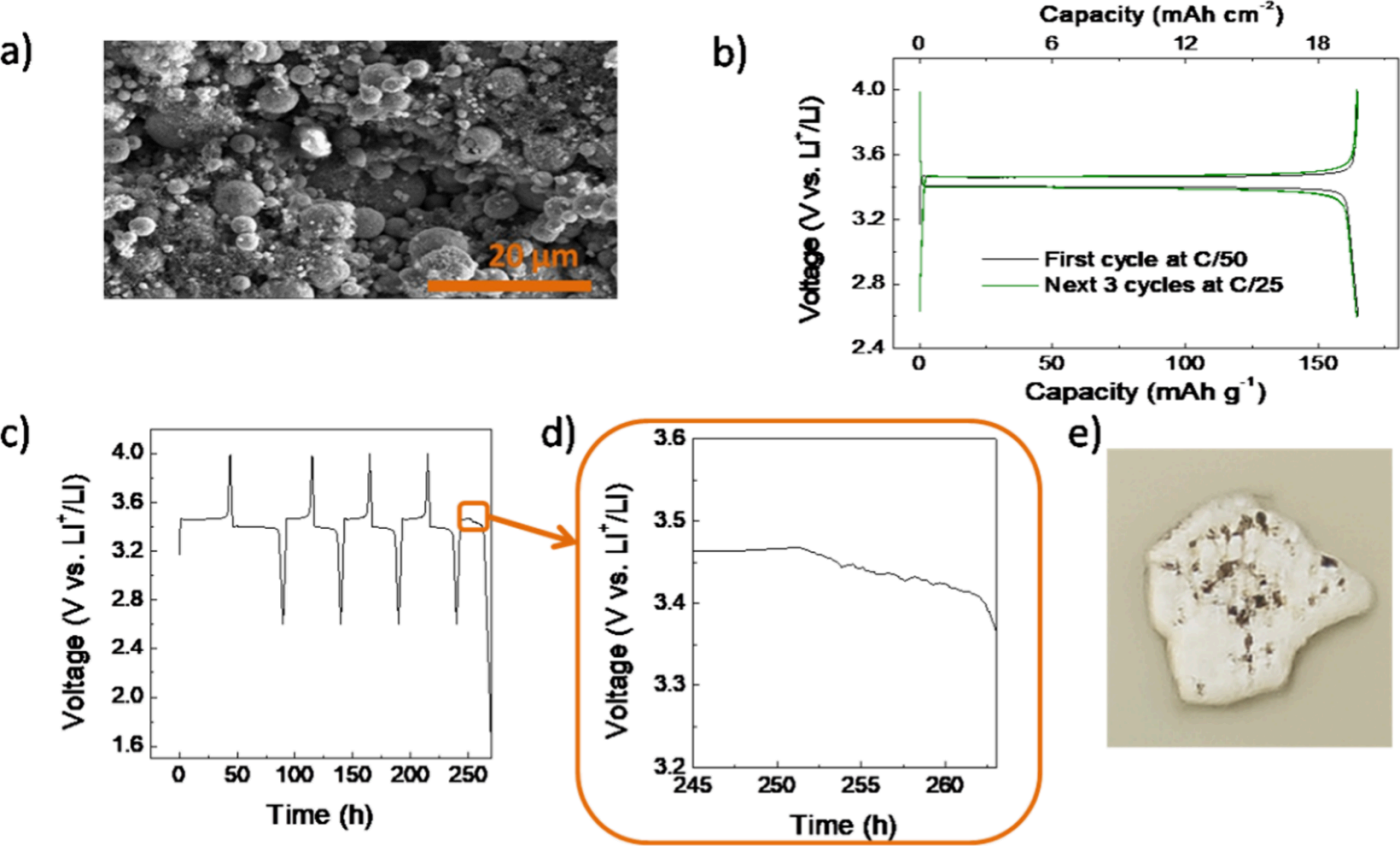
(a) SEM image of the cross-section of the electrode, (b) voltage profile *vs.* capacity for the first cycle at C/50 and for the three subsequent cycles at C/25 for a LFP thick electrode–metallic Li cell, (c) voltage profile of the same cell over time, (d) magnified view of the time period where dendritic effects are observed in the voltage profile, and (e) picture of the recovered separator piece damaged due to dendrite formation.

To avoid dendrite formation and demonstrate the real applicability of these thick electrodes, CNT-LTO electrodes were developed following the same procedure. To ensure an excess of the counter electrode to avoid LFP acting as the limiting electrode, coin cells were assembled using double the amount of CNT-LTO relative to LFP. Figure 3 summarizes the main electrochemical results of the LFP–LTO cells. The first charge for the formation cycle at C/50 delivers 161 mAh g^–1^, similar to that obtained in the half-cell, with an irreversibility of 12.5% still delivering 141 mAh g^–1^. This irreversible capacity has been observed in association with the presence of CNTs, mainly due to their high surface area that promotes SEI formation and lithium trapping in their porous structure.^25^ Despite this initial loss, CNTs enhance cycling performance by improving electrical conductivity and ion diffusivity.^25,26^ In the next cycle at C/25, the cell is able to keep the same capacity, and this capacity is only reduced by 7% when increasing the rate to C/6.25. 112 mAh g^–1^ is still displayed at C/4. This loss of capacity in very thick electrodes is mainly due to ion and electron transport limitations, which lead to nonuniform reactions across the electrode. As a result, most of the electrochemical activity takes place near the surface, causing partial use of the active material and a lower apparent capacity.^27^

**Figure 3:**
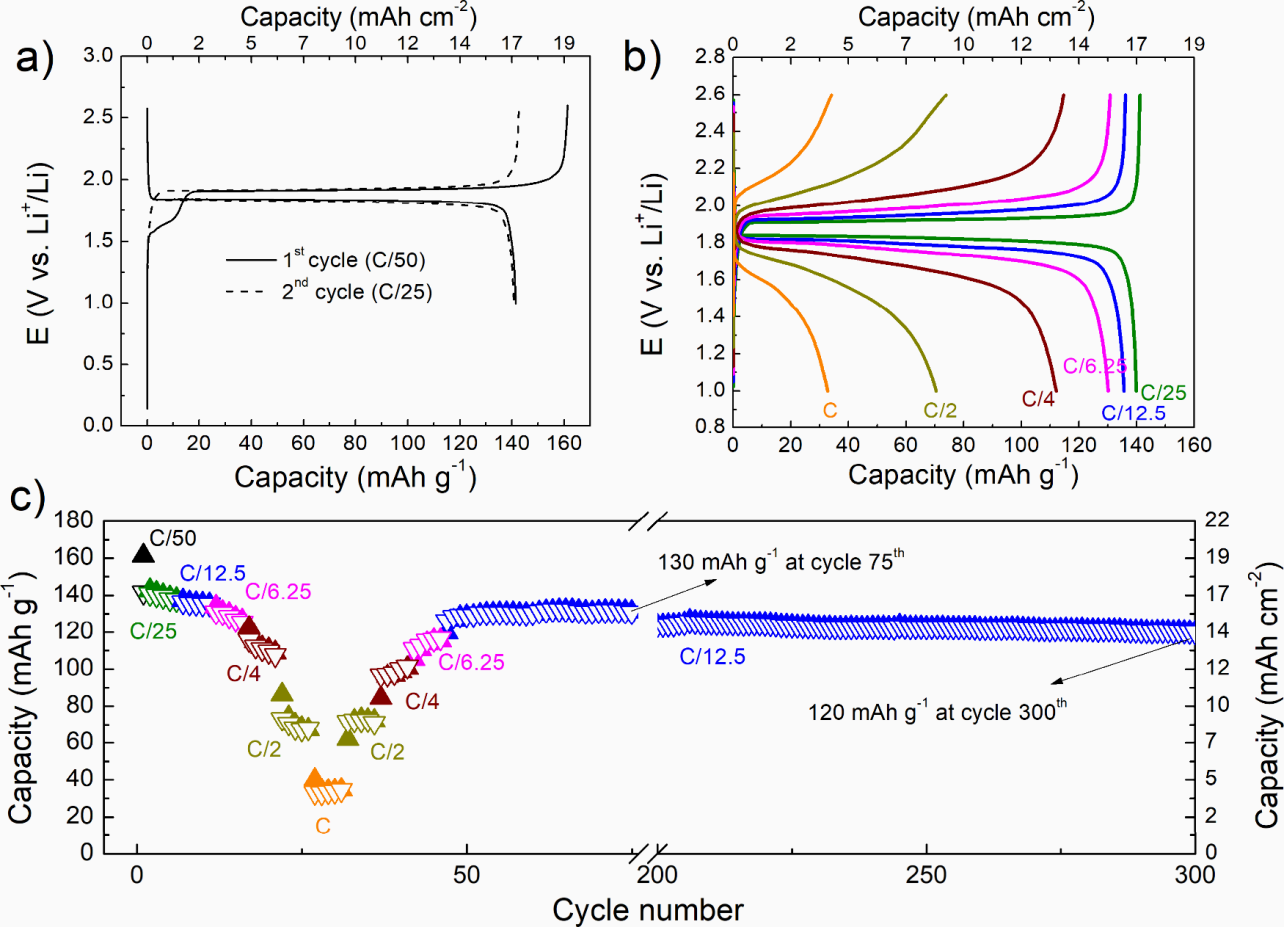
(a) Voltage profile for the first and second cycles at C/50 and C/25, respectively, for LFP-LTO cells, (b) charge–discharge voltage profile at different C-rates for thick electrodes (650 μm and 120 mg cm^–2^ of LFP and 113 mg cm^–2^ of LTO), and (c) gravimetric specific capacity vs cycle number at different C-rates for the LFP-LTO cell configuration with thick electrodes.

More interestingly, the high areal capacities obtained are 17 and 13.5 mAh cm^–2^ at C/25 and C/4, respectively. At the highest rates, the gravimetric capacity decreased to 70 and 33 mAh g^–1^, but in terms of areal capacity, they are still 8.5 and 4 mAh cm^–2^. These values clearly overcome those of typical commercial electrodes with thicknesses usually lower than 100 μm (∼8 mg cm^–2^ and 1 mAh cm^–2^).^28^

The porosity of the electrodes is an important parameter for battery performance, as it affects not only electronic and ionic transport within the electrode (percolation) but also mechanical properties such as the elastic modulus and brittleness.^29–33^ In our case, apart from the electronic conductive properties kept due to the use of C65 additive,^34^ the excellent rate capability of these electrodes can also be attributed to their high porosity (∼45%), allowing a lower tortuosity for the electrolyte impregnation, increasing the ionic conductivity. This improved electrode–electrolyte contact also enhances the charge-transfer kinetics, as also reported for thick sintered LFP electrodes (∼1 mm) that showed much better performance and lower overpotential when the porosity was 44% compared to 22%.^7^

To the best of our knowledge, the cycling performance in this work surpasses other works using composite thick electrodes vs LTO as counter electrode and common carbonate electrolytes. Hu et al. showed an LFP/LTO cell with their textile electrodes of 145 mg cm^–2^, 500 μm thickness, and high areal capacity (21 mAh cm^–2^ at C/10) but a decrease in capacity of 11.5% after 30 cycles.^35^ Wu et al., using thick electrodes of 1000 μm, obtained areal capacities of 14.4 mAh cm^–2^ for the LCO/LTO cell, but a decay in capacity of 32% after 30 cycles.^21^ In this work, after the rate capability, the capacity stabilized at 130 mAh g^–1^ (15.6 mAh cm^–2^) at C/12, decreasing by less than 8% at the 300th cycle. Maintaining PVDF as a binder on the thick electrode, known for its mechanical strength and corrosion resistance, may contribute significantly to the high cycling stability.^36^

## Conclusions

Thick self-standing composite electrodes (650 μm and 120 mg cm^–2^) containing PVDF and C65 as additives have been successfully developed by a simple modification of the traditional method, which involves drying the NMP prior to the hot pressing of the mixed components. The full cell assembled with these electrodes demonstrated excellent performance across a wide range of charge/discharge rates, achieving high areal capacities. Moreover, its cycling stability surpasses previously reported values for similar systems. Specifically, the cell delivered 16.3 mAh cm^–2^ at C/12 for 300 cycles, retaining over 92% of its initial capacity. The simplicity of the fabrication method requires only minimal adjustments to the conventional Li-ion electrode manufacturing processes, making it a viable and scalable approach for the development of next-generation batteries with a higher energy density.

## Supplementary Material



## Notes

An image of the microstructure of the prepared electrodes (Figure S1), plot of conductivity values vs thickness (Figure S2), and voltage profile during charge–discharge process of LFP/Li half-cells with the discharge cutoff voltage restricted to 3.0 V (Figure S3) (PDF)
