# Supplementary material for: A Simple Hot-Pressing Strategy for Thick Lithium Iron Phosphate Electrodes with Outstanding Electrochemical Properties
Source: ACS Omega. 2025 Dec 29;11(1):681–6. doi: 10.1021/acsomega.5c06687 (PMC12809511; doi:10.1021/acsomega.5c06687)
Supplement: Supplementary file 1 [file ao5c06687_si_001.pdf]

Supplementary Information

A Simple Hot-Pressing Strategy for Thick LFP Electrodes with Outstanding Electrochemical Properties

*Antonio J. Fernández-Ropero, Daniel del Rio-Santos, Belén Levenfeld and Alejandro Varez**

Universidad Carlos III de Madrid, Department of Materials Science and Engineering and Chemical Engineering, IAAB, Leganes, 28911, Madrid, Spain

*Corresponding author.
E-mail address: alvar@ing.uc3m.es (A. Varez)

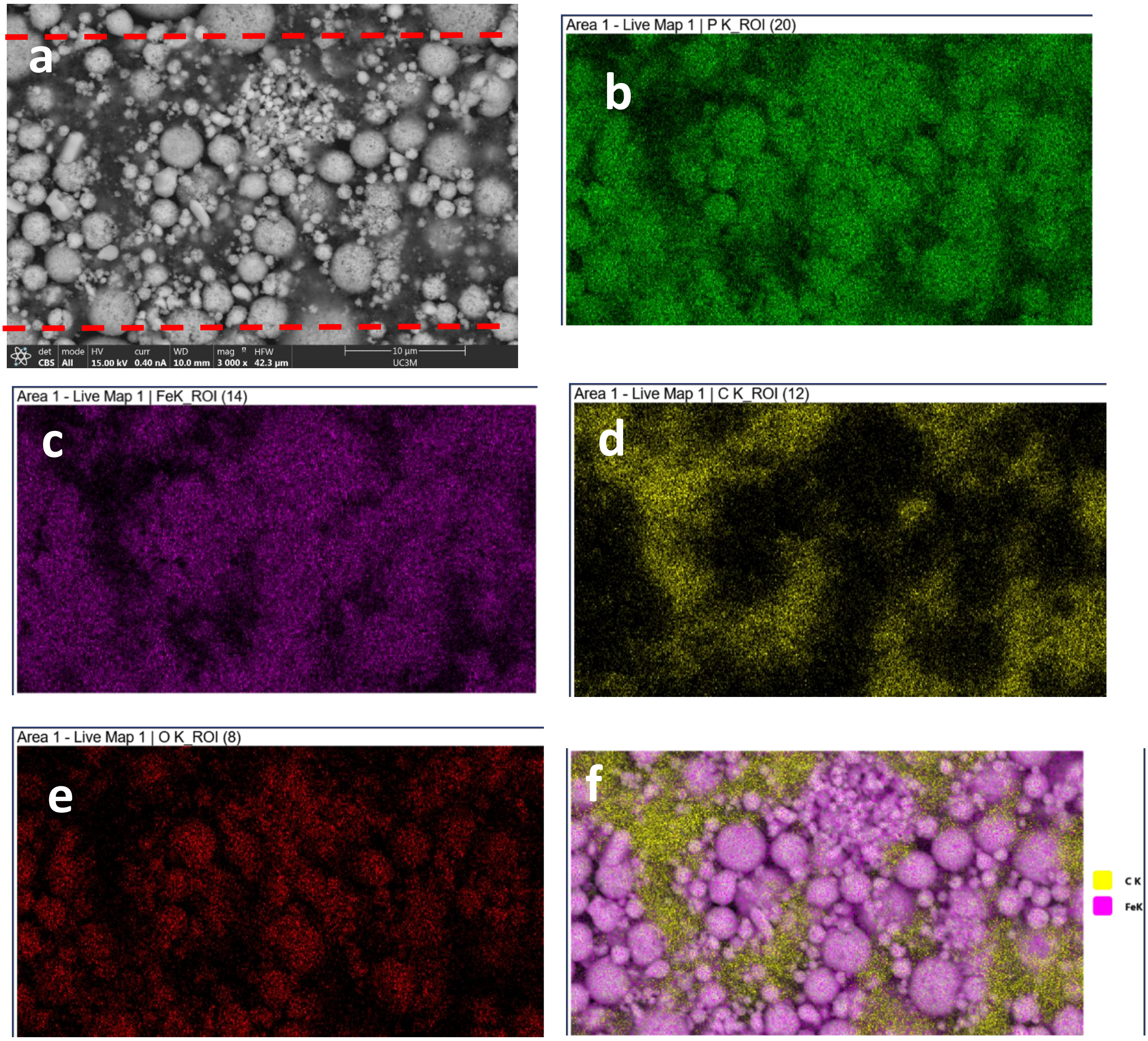

Figure SI-1. *(a) SEM image of the LFP electrode. Bright spherical features correspond to LFP particles, while the grey contrast is associated with the PVDF + C binder surrounding the active material, and the black contrast indicates porous regions. (b–e) Elemental X-ray mapping showing the distribution of Fe, C, O, and P across the sample. (f) Overlay of Fe and C elemental maps on the SEM image.*

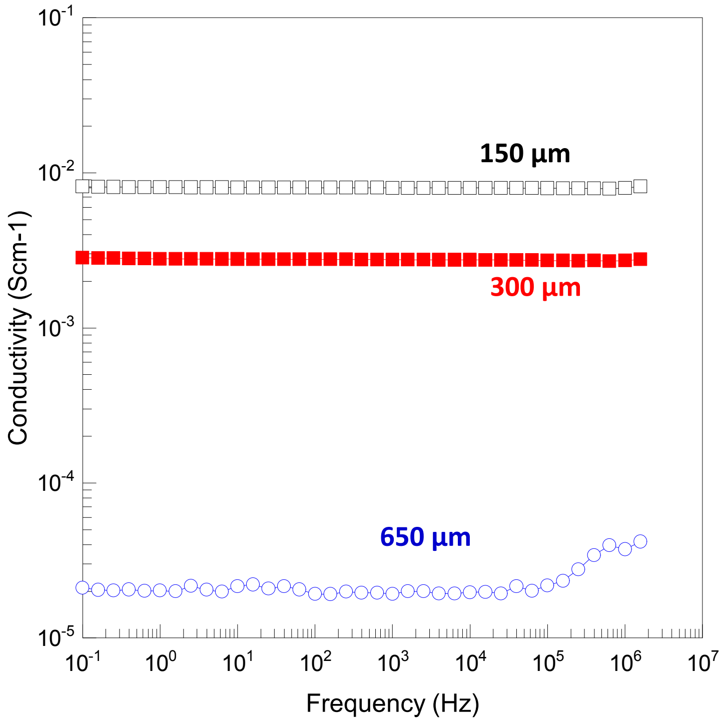

Figure SI-2.- *Variation of the conductivity as a function of frequency for electrodes with thicknesses of 150, 300, and 650 µm. The conductivity spectra display a frequency-independent plateau region, characteristic of electronic conduction in percolated networks. The absolute value of conductivity decreases with increasing electrode thickness, which can be attributed to the combined effect of higher porosity and extended electron transport paths. Despite this reduction, the flat frequency response confirms that the conductive additive forms an efficient and continuous electronic network across the electrode thickness.*

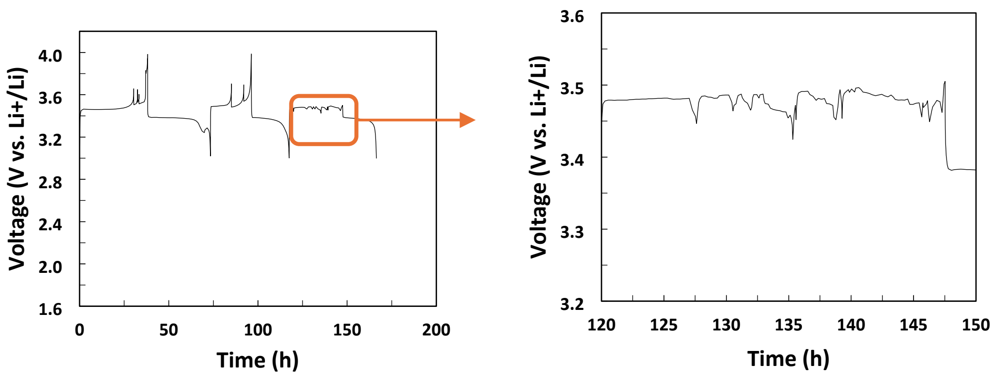

Figure SI-3.-*Voltage profile during charge-discharge process of LFP/Li half-cells with the discharge cut-off voltage restricted to 3.0 V. Despite the narrower cycling window, dendritic growth and cell short-circuit were still observed, confirming that the phenomenon is mainly associated with Li deposition during charging rather than with the discharge depth.*
